# Extracellular matrix protein laminin enhances mesenchymal stem cell (MSC) paracrine function through αvβ3/CD61 integrin to reduce cardiomyocyte apoptosis

**DOI:** 10.1111/jcmm.13087

**Published:** 2017-06-09

**Authors:** Kai‐Yen Peng, Yuan‐Hung Liu, Yu‐Wei Li, Betty Linju Yen, Men‐Luh Yen

**Affiliations:** ^1^ Department of Life Science National Central University Jhongli Taiwan; ^2^ Regenerative Medicine Research Group Institute of Cellular & System Medicine National Health Research Institutes Zhunan Taiwan; ^3^ Section of Cardiology Cardiovascular Center Far Eastern Memorial Hospital Pan Chiao, New Taipei City Taiwan; ^4^ Department of Obstetrics/Gynecology National Taiwan University Hospital and College of Medicine National Taiwan University Taipei Taiwan

**Keywords:** human mesenchymal stem cells (MSCs), extracellular matrix (ECM), laminin, integrin, apoptosis, cardiomyocyte

## Abstract

Myocardial ischaemia (MI) results in extensive cardiomyocyte death and reactive oxygen species (ROS)‐induced damage in an organ with little or no regenerative capacity. Although the use of adult bone marrow mesenchymal stem cells (BMMSCs) has been proposed as a treatment option, the high cell numbers required for clinical use are difficult to achieve with this source of MSCs, and animal studies have produced inconsistent data. We recently demonstrated in small and large animal models of acute MI that the application of human term placenta‐derived multipotent cells (PDMCs), a foetal‐stage MSC, resulted in reversal of cardiac injury with therapeutic efficacy. However, the mechanisms involved are unclear, making it difficult to strategize for therapeutic improvements. We found that PDMCs significantly reduced cardiomyocyte apoptosis and ROS production through the paracrine factors GRO‐α, HGF and IL‐8. Moreover, culturing PDMCs on plates coated with laminin, an extracellular matrix (ECM) protein, resulted in significantly enhanced secretion of all three paracrine factors, which further reduced cardiomyocyte apoptosis. The enhancement of PDMC paracrine function by laminin was mediated through αvβ3 integrin, with involvement of the signalling pathways of JNK, for GRO‐α and IL‐8 secretion, and PI3K/AKT, for HGF secretion. Our results demonstrated the utility of PDMC therapy to reduce cardiomyocyte apoptosis through modulation of ECM proteins in *in vitro* culture systems as a strategy to enhance the therapeutic functions of stem cells.

## Introduction

Ischaemic heart disease (IHD) is a leading cause of death worldwide 1. Atherosclerosis, the thickening and hardening of the coronary arteries resulting from the deposition of lipids in vessel walls, is the most common cause of MI, a type of IHD. When the blood supply is not quickly restored following coronary artery occlusion, the surrounding cardiac tissue undergoes necrosis and apoptosis 2, 3. Current treatment options consist of surgical repair to increase the blood supply, but once the myocardium is damaged, it is replaced by fibrosis and cannot be regenerated. This can result in permanent cardiac dysfunction and lead to death. Stem cell therapy performed with bone marrow (BM) mononuclear cells and stem cells – including mesenchymal stem cells (MSCs) – has been proposed for the treatment of IHD, but clinical results have not been as robust as those seen in animal studies 4, 5. This may be due to a number of factors, including cell volume used, method of cell delivery and post‐delivery cell survival 6. Moreover, these adult progenitors are quite rare, and cell numbers further decrease with increasing age 7. Because IHD incidence increases with age, the use of any type of autologous BM stem cell would lose efficacy as the patient ages.

We have recently shown that human term PDMCs – a type of MSC with a foetal origin 8 – can modulate cardiac injury after MI induction in both small and large animal models 9. Interestingly, the transplantation of PDMCs led to more significant improvements in cardiac function in the large animal model (pig) than in the small animal model (mouse). In addition to enhancing angiogenesis, as was seen in the mouse model, we found that PDMCs also reduced cardiomyocyte apoptosis in the pig model. Because cardiomyocyte death is the key pathologic reason for compromised cardiac function in IHD, we are interested in exploring the mechanisms involved in the anti‐apoptotic effects of PDMCs. In this study, we established an *in vitro* system to mimic key events in MI‐induced cardiomyocyte apoptosis using tumour necrosis factor‐α (TNF‐α), a key molecule involved in MI‐induced cardiac injury. TNF‐α is a multifunctional cytokine that promotes cell survival or death depending on its dose and context and is found at levels eightfold to 10‐fold above normal in the infarct and border zones of the post‐MI heart 10. High levels of TNF‐α result in not only cardiomyocyte apoptosis but also production of ROS, which lead to further cell injury and death 11, 12. To enhance the effects of PDMCs, we have modulated the *in vitro* cell culture conditions with the use of ECM proteins, which are known to be an important part of the stem cell microenvironment/niche and to regulate stem cell differentiation, proliferation, migration and function 13. The *in vitro* use of specific ECM proteins has been shown to modulate stem cell differentiation capacity and lineage commitment 14, 15, 16. However, it has not been reported whether ECM proteins modulate stem cell paracrine factor production in *in vitro* cell culture. Our data in this study demonstrated that the paracrine function of PDMCs could be enhanced by the ECM protein laminin through αvβ3 integrin/CD61, an integrin that is highly expressed on MSCs 16, 17 and to which laminin has high affinity 18. Our findings thus can provide strategies to improve the therapeutic use of MSCs for the treatment of IHD.

## Materials and methods

### Cell culture and related experiments

Term human placenta tissue samples (38‐ to 40‐week gestation) from healthy donor mothers were obtained with informed consent and approval according to the procedures of the institutional review board. PDMCs were isolated and expanded as previously described 8. Briefly, placental tissue was mechanically and enzymatically dissected and cultured in complete medium consisting of Dulbecco's modified Eagle's medium (DMEM; Gibco‐Invitrogen, Carlsbad, CA, USA) supplemented with 10% foetal bovine serum (FBS; selected lots, Hyclone, Thermo Scientific, Waltham, MA, USA). When cells were at 80–90% confluence, they were subcultured at a dilution of 1:3. WS1, human foetal skin fibroblasts, were purchased from Bioresource Collection and Research Center (BCRC, Hsinchu, Taiwan) and were cultured as recommended in minimum essential medium (MEM; Gibco‐Invitrogen) with 10% FBS (Gibco‐Invitrogen). Mouse cardiomyocytes from 8‐week‐old C57BL/6 mice were isolated as previously described 19, cultured in DMEM (Gibco‐Invitrogen) with 10% FBS (Gibco‐Invitrogen), and characterized for expression of the cardiomyocytic markers cardiac troponin‐T, Nkx2.5 and connexin43 20. For experiments with recombinant proteins, recombinant human proteins (all obtained from Peprotech, Rocky Hill, NJ, USA) were added to cell cultures at the indicated doses. For experiments involving coating with various ECM proteins, six‐well plates were either not coated [indicated by the addition of phosphate‐buffered saline (PBS)] or coated with ECM gel (50 μg/ml; Sigma‐Aldrich, St Louis, MO, USA), gelatin (0.4%, Sigma‐Aldrich), laminin (50 μg/ml; Invitrogen, Waltham, MA, USA), collagen type I (50 μg/ml; BD Biosciences, Sparks, MD, USA) or fibronectin (50 μg/ml; Sigma‐Aldrich). Per the manufacturer's instructions, ECM gel was isolated from Engelbreth‐Holm‐Swarm (EHS) sarcoma generated in mice, and it contained laminin, collagen type IV, heparan sulphate proteoglycan, entactin and other minor components. All culture plate/well coating was performed by applying a solution containing the indicated protein/gel to culture plates/wells and incubating at 37°C for 2 hrs, then aspirating the solution from the plates/wells, and allowing them to air dry. Conditioned medium (CM) was collected from cell cultures after 48 hrs of culturing. Signal pathway inhibitors (and concentrations) used are as follows: MK2206 (AKT inhibitor, 10 μM; Cell Signaling Technology, Danvers, MA, USA); LY294002 (PI3K inhibitor, 10 μM; Cell Signaling Technology); SB203580 (p38 inhibitor, 20 μM; Merck & Co., Kenilworth, NJ, USA); SP600125 (JNK inhibitor, 20 μM; Cell Signaling Technology); U0126 (MEK1/2 inhibitor, 20 μM; Cell Signaling Technology); PD98059 (MEK‐1 inhibitor, 20 μM; Cell Signaling Technology); and Cpd188 (STAT3 inhibitor, 5 μM; Merck & Co.) for 48 hrs.

### Apoptosis assay

Cardiomyocytes were cultured on six‐well plates (1.5 × 10^5^ cells per well) and treated with TNF‐α (20 ng/ml; Peprotech) alone or with recombinant human growth‐regulated oncogene‐α (GRO‐α; 50 ng/ml), hepatocyte growth factor (HGF; 20 ng/ml), interleukin‐6 (IL‐6; 10 ng/ml) or interleukin‐8 (IL‐8; 50 ng/ml) for 48 hrs. Cardiomyocytes were assayed for apoptosis with the Annexin V‐FITC Apoptosis Detection kit (eBioscience, San Diego, CA, USA). Cells were harvested by centrifugation (1200 × *g*, 5 min.), after which the medium was discarded. After washing with PBS, cells were recovered and stained with FITC‐conjugated annexin V and propidium iodide (PI) for 20 min. Apoptotic cardiomyocytes were detected by flow cytometry (BD Biosciences). Cells (1 × 10^4^ cells per sample) were counted, and each sample was analysed with WINMDI software (Purdue University Cytometry Laboratories, Purdue University, West Lafayette, IN, USA). All experiments were repeated at least three times.

### Enzyme‐linked Immunosorbent Assay (ELISA)

CM from cells cultured in various conditions (*i.e*. on different ECM protein‐coated plates, after RNAi knockdown) was collected, and the levels of human GRO‐α, HGF, IL‐6 and IL‐8 were measured by enzyme‐linked immunosorbent assay (ELISA). ELISA kits for GRO‐α, HGF and IL‐8 were obtained from R&D systems (Minneapols, MN, USA), and that for IL‐6 was obtained from eBioscience. The experimental procedures were executed according to the manufacturer's instructions.

### ROS measurement

Cells were cultured on six‐well plates (1.5 × 10^5^ cells per well) and treated with TNF‐α (20 ng/ml) alone or with GRO‐α (50 ng/ml), HGF (20 ng/ml) or IL‐8 (50 ng/ml) for 48 hrs. To measure intracellular ROS, cells were stained with 2′, 7′‐dichlorofluorescein diacetate (DCFDA, 20 μM; Sigma‐Aldrich) for 30 min. and then washed with PBS. ROS levels were determined by measuring dichlorofluorescein (DCF) fluorescence with flow cytometry. All experiments were repeated at least three times.

### Western blotting

Western blotting was performed as previously reported 16. Briefly, mouse cardiomyocytes were lysed in protein extraction reagent (Thermo Scientific). Total proteins were separated by electrophoresis on a 15% SDS–polyacrylamide gel and transferred to a nitrocellulose membrane. The membranes were blotted with caspase‐3 antibody (Cell Signaling Technology). After washing with PBS Tween‐20, the membranes were incubated with horseradish peroxidase (HRP)‐conjugated secondary antibody for 1 hr at room temperature. Signals were detected with chemiluminescent HRP substrate (Millipore, Billerica, MA, USA).

### RNA isolation and Reverse transcription–polymerase chain reaction (RT‐PCR)

RT‐PCR was performed as previously reported 21. Briefly, total RNA was isolated from cardiomyocytes performed with TRIzol (Invitrogen) and reverse‐transcribed to cDNA performed with the ImProm‐ll Reverse Transcriptase system (Promega, Madison, WI, USA). Antioxidant genes were detected by PCR. The following primers were used: mouse catalase, forward primer 5ʹ‐GGACAGTCGGGACCCAGCCA‐3ʹ, reverse primer, 5ʹ‐ CGACTGTGGAGAACCGAACGGC‐3ʹ; mouse CuZnSOD, forward primer 5ʹ‐ AGAAACATGGTGGCCCGGCG‐3ʹ, reverse primer, 5ʹ‐ CTCCACAGGCCAAGCGGCTC‐3ʹ; and β‐actin positive control, forward primer 5ʹ‐TGGCACCACCTTCTACAATGAGC‐3ʹ, reverse primer 5ʹ‐ GCACAGCTTCTCCTTAATGTCACGC‐ 3ʹ.

### RNA interference

Small interfering RNA (siRNA) specific for αvβ3 integrin or the non‐target control (Gibco‐Invitrogen) was transfected into PDMCs according to the manufacturer's recommendations and as previously reported 16. Cells were seeded on six‐well plates (1.5 × 10^5^ cells/well) and then treated with transfection reagent and either siRNA specific for αvβ3 integrin or non‐target RNA. After 48 hrs, the expression levels of αvβ3 integrin in transfected cells were confirmed by RT‐PCR.

### Statistical analysis

All experiments were performed at least in triplicate, and the data are presented as the mean ± S.E.M. Both parametric analyses, Student's *t‐*test for comparisons of two variables and anova for comparisons of more than two variables, and nonparametric analyses, the Mann–Whitney *U*‐test for comparisons of two variables and Kruskal–Wallis test for comparisons of more than two variables, were performed. A value of *P* < 0.05 was defined as statistically significant.

## Results

### PDMC‐CM reduces the number of apoptotic cardiomyocytes

**Figure 1:**
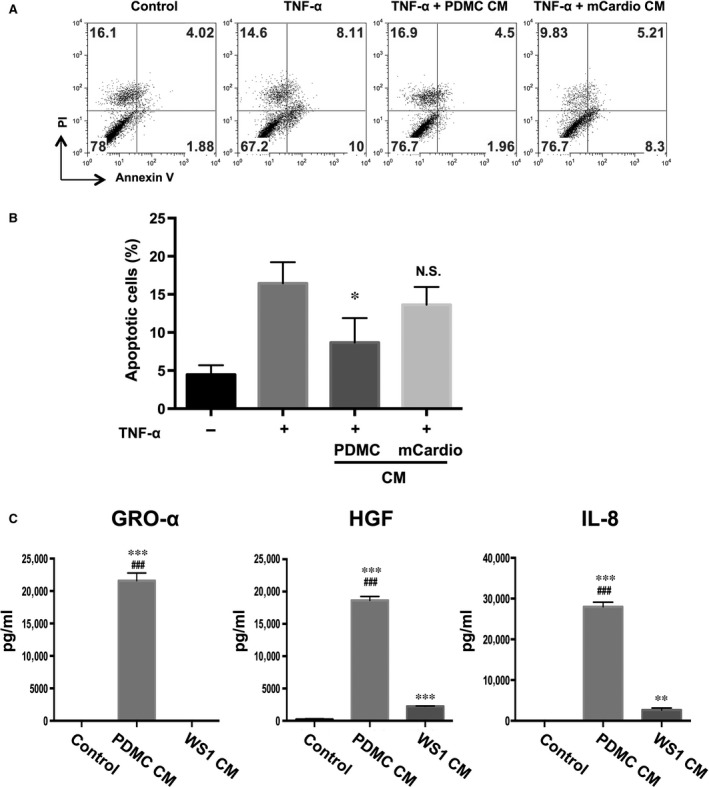
Placenta‐derived multipotent cell (PDMC)‐conditioned medium (CM) reduces the percentage of apoptotic cardiomyocytes. (**A**) PDMC‐CM can reduce mouse cardiomyocyte apoptosis. Mouse cardiomyocytes treated with TNF‐α (20 ng/ml) alone or mixed with PDMC‐CM or mouse cardiomyocyte CM (mCardio‐CM) were stained for annexin V and propidium iodine (PI) and analysed by flow cytometry. Representative graphs and quantitative results (**B**) are shown and presented as the mean ± S.E.M. of three independent experiments. (**C**) PDMCs secrete high levels of GRO‐α, HGF and IL‐8. ELISA was used to detect GRO‐α, HGF and IL‐8 in PDMC‐CM and fibroblast (WS1) CM. All results are shown as the mean ± S.E.M. of three independent experiments. *P < 0.05; **P < 0.01; and ***P < 0.005 compared to the control; ###*P <* 0.005 compared to WS1‐CM.

Our previous work showed that PDMCs could ameliorate MI‐induced cardiomyocyte apoptosis, likely through secreted factors. To ascertain whether these anti‐apoptotic effects of PDMCs are mediated by secreted factors, we added PDMC‐CM to TNF‐α‐treated cardiomyocytes (1:1 mixed with cultured medium). After 48 hrs, TNF‐α induced apoptosis in approximately 18.1% of treated cells, as demonstrated by positive staining for annexin V, a marker for apoptosis (Fig. 1A). The addition of PDMC‐CM to TNF‐α‐treated cardiomyocytes reduced the percentage of apoptotic cells down to 6.5%, whereas the addition of CM from mouse cardiomyocytes (mCardio‐CM) only reduced the percentage to 13.5%. The quantitative results showed PDMC‐CM but not mCardio‐CM significantly reduced the percentage of apoptotic cells from an average of 16.5% down to 8.7% (Fig. 1B). Our results indicate that PDMC paracrine factors suppress TNF‐α‐induced cardiomyocyte apoptosis.

### High levels of GRO‐α, HGF and IL‐8 but not IL‐6 secreted by PDMCs are involved in reducing TNF‐α‐induced apoptosis in cardiomyocytes

**Figure 2:**
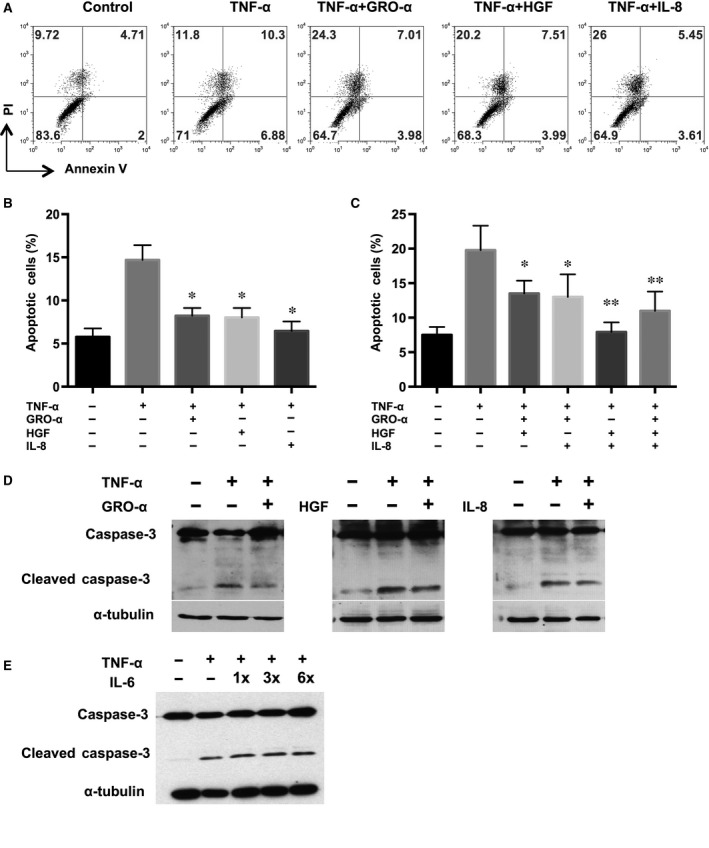
GRO‐α, HGF and IL‐8 reduced the percentage of apoptotic cardiomyocytes. (**A**) mCardios were treated with TNF‐α only or with the addition of recombinant GRO‐α, HGF, and/or IL‐8 (**B**) alone or (**C**) in combination. After 48 hrs, mCardios were stained for annexin V and PI and analysed by flow cytometry. (**D**) Activated/cleaved caspase‐3 in TNF‐α‐treated mCardios was assayed by Western blotting after the addition of recombinant GRO‐α, HGF, IL‐8 or (**E**) IL‐6. α‐Tubulin was used as an internal control. All results are shown as the mean ± S.E.M. of three independent experiments. *P < 0.05 and **P < 0.01 compared to TNF‐α only.

We have previously shown using qualitative cytokine arrays that PDMCs secrete high levels of GRO‐α, HGF and IL‐8, which are involved in modulating ischaemic cardiac injury 9. To quantify the secreted levels of these three factors by PDMCs, we performed ELISA and found that PDMCs secrete higher levels of all three factors than WS1 human fibroblasts (Fig. 1C). To test whether these three PDMC‐secreted factors are directly involved in anti‐apoptotic effects, we induced cardiomyocyte apoptosis with TNF‐α and treated them with GRO‐α, HGF and IL‐8 recombinant proteins alone or in combination, and we found that all three of these proteins significantly reduced the percentage of apoptotic cells, with IL‐8 showing the strongest effect from 17.2% to 9.1% (Fig. 2A and B). Furthermore, treatment with the combination of these factors appeared to have more potent anti‐apoptotic effects than treatment with any factor alone, with HGF/IL‐8 reducing apoptotic levels more effectively than HGF/GRO‐α or GRO‐α/IL‐8 (Fig. 2C). These effects are specific to these secreted factors because the addition of recombinant TIMP‐1 and TIMP‐2, two factors that are highly secreted by both PDMCs and BMMSCs 9, did not reduce TNF‐α‐induced apoptosis (data not shown). To ascertain whether GRO‐α, HGF and IL‐8 reduced the number of apoptotic cells through caspase‐3, a critical mediator of apoptosis, we detected the activation of caspase‐3 in TNF‐α‐induced apoptotic cardiomyocytes treated with GRO‐α, HGF or IL‐8 recombinant proteins. Interestingly, GRO‐α, HGF and IL‐8 reduced the formation of the active form of caspase‐3 induced by TNF‐α in cardiomyocytes (Fig. 2D). To assess the specificity of these effects, we administered IL‐6 (10, 30 and 60 ng/ml) – which is known to be highly secreted by MSCs and other stromal cells – to TNF‐α‐induced apoptotic cardiomyocytes and found no suppression of caspase‐3 activation (Fig. 2E). Thus, our results indicate that GRO‐α, HGF and IL‐8 suppress cardiomyocyte apoptosis, with IL‐8 having stronger effects than GRO‐α and HGF.

### GRO‐α, HGF and IL‐8 reduce the level of TNF‐α‐induced ROS

**Figure 3:**
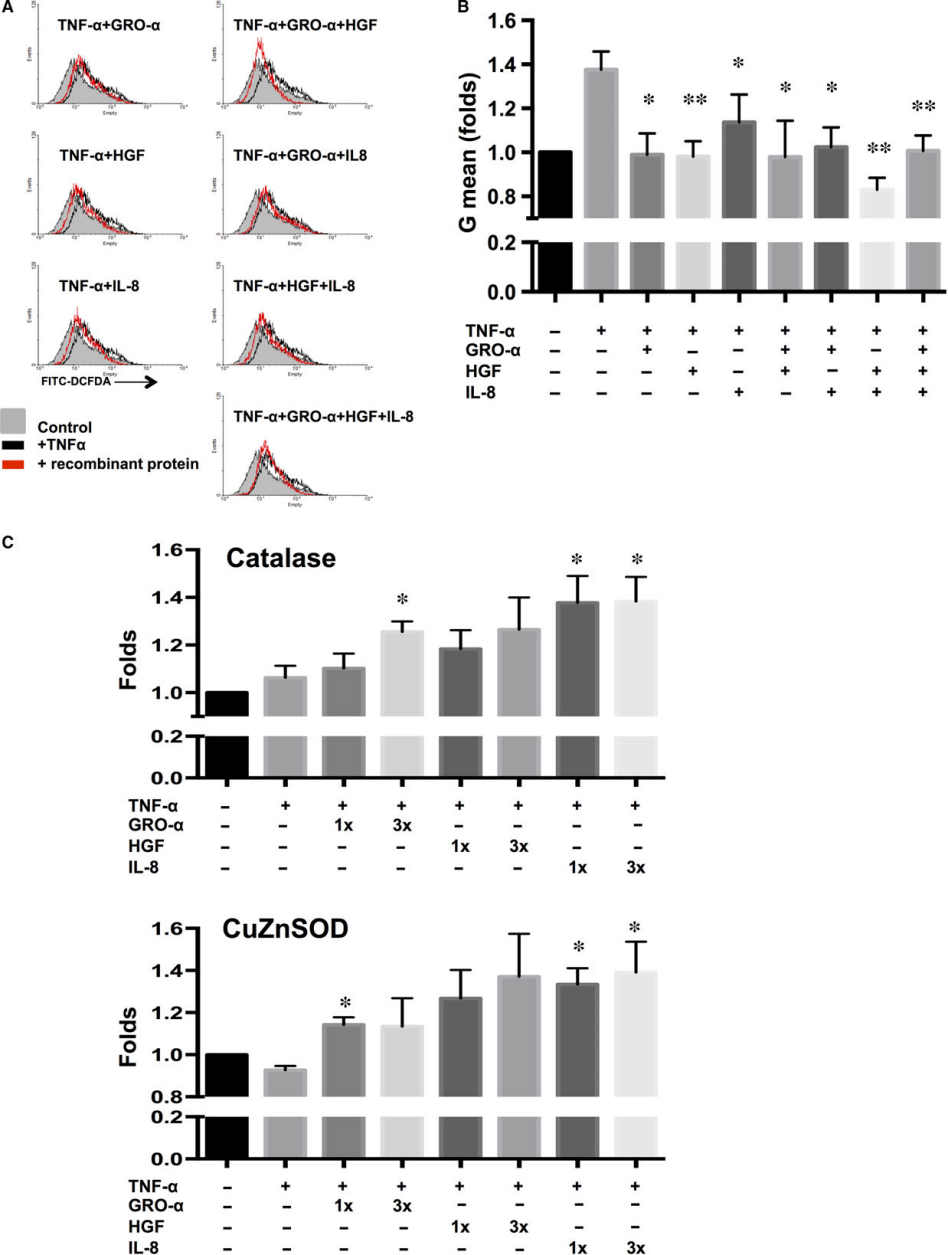
GRO‐α, HGF and IL‐8 reduced the levels of TNF‐α‐induced ROS in mCardios. (**A**) mCardios were treated with TNF‐α alone or with the addition of recombinant GRO‐α, HGF and/or IL‐8, and ROS levels were measured using the dye 2′, 7′‐dichlorofluorescein diacetate (DCFDA) followed by analysis by flow cytometry. Representative figures and (**B**) quantitative results are shown. (**C**) The gene expression of *Catalase* and *CuZnSOD* in mCardios treated with TNF‐α alone or with the addition of recombinant GRO‐α, HGF, and/or IL‐8 was analysed by RT‐PCR with quantification. All results are shown as the mean ± S.E.M. of three independent experiments. *P < 0.05 and **P < 0.01 compared to TNF‐α only.

ROS are known to be involved in cardiomyocyte apoptosis, so we assessed whether GRO‐α, HGF and IL‐8 affect ROS production in TNF‐α‐stimulated cardiomyocytes. Cardiomyocytes were treated with these three proteins alone or in combination, and then, intracellular ROS levels were measured performed with H_2_DCFDA staining. We found that TNF‐α induced ROS production in cardiomyocytes, which could be reduced by any of the three factors, with the strongest effects seen when either GRO‐α or HGF were added or when the combination of HGF/IL‐8 was used (Fig. 3A and B). To further elucidate the suppressive effects of these paracrine factors on ROS production, we assayed for the expression of the antioxidant‐associated genes *Catalase* and *CuZnSOD* and found that the addition of GRO‐α, HGF and IL‐8 increased the expression of *Catalase* and *CuZnSOD* in TNF‐α‐stimulated cardiomyocytes (Fig. 3C). Our results demonstrate that PDMC‐secreted factors, particularly HGF, enhance the expression of antioxidant enzymes and modulate TNF‐α‐induced ROS production in cardiomyocytes.

### Laminin enhances PDMC secretion of GRO‐α, HGF and IL‐8

**Figure 4:**
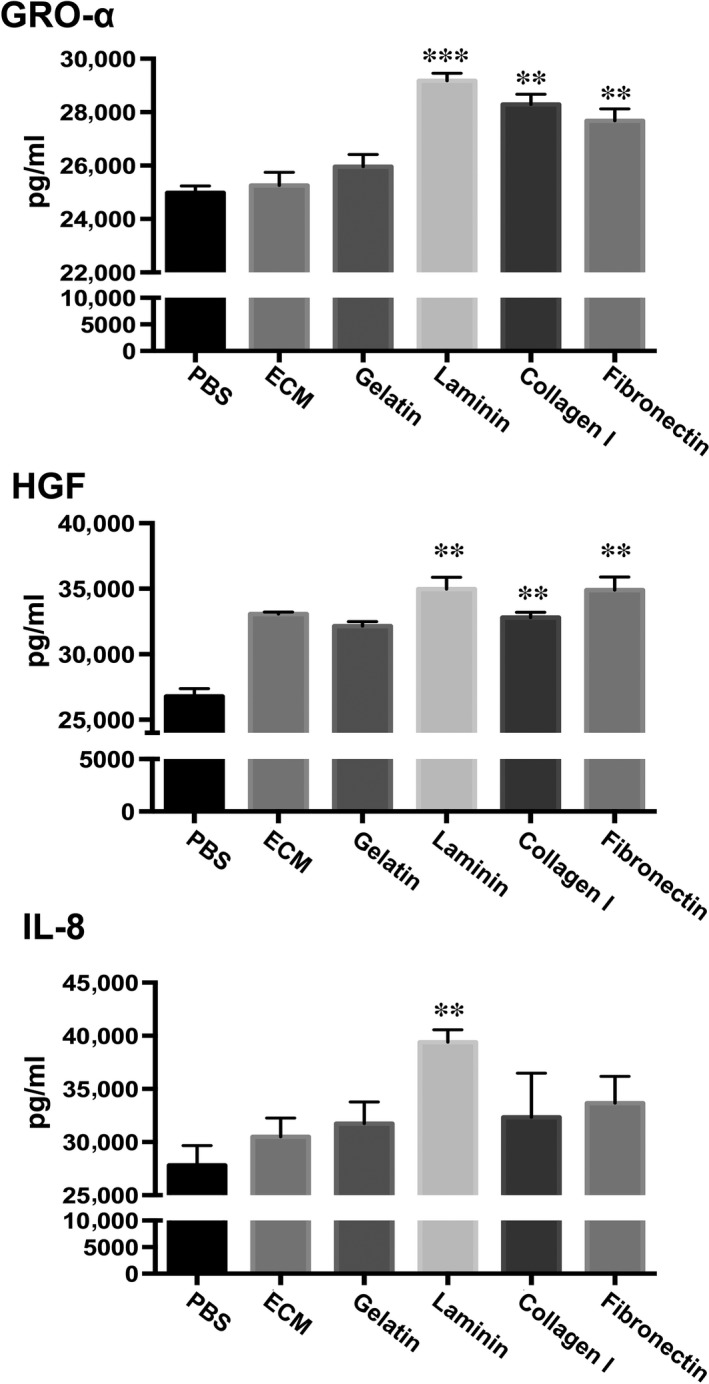
PDMCs cultured on laminin exhibit enhanced secretion of GRO‐α, HGF and IL‐8. PDMCs were cultured on uncoated plates (PBS) or plates coated with ECM proteins as indicated. After 48 hrs, CM was collected and analysed for GRO‐α, HGF and IL‐8 with the appropriate ELISA kit, and the values were normalized with cell numbers. All results are shown as the mean ± S.E.M. of three independent experiments. **P < 0.01 and ***P < 0.005 compared to PBS.

To better mimic the *in vivo* setting and assess whether paracrine functions can be modulated, we cultured PDMCs on plates that were coated with ECM proteins, including mixtures such as ECM gel and gelatin, or specific proteins such as laminin, collagen type I and fibronectin. We found that the ECM protein laminin promoted the secretion of all three factors. Culturing on laminin on average enhanced PDMC secretion of GRO‐α from 25.0 to 29.2 ng/ml, HGF from 26.8 to 35.0 ng/ml and IL‐8 from 27.8 to 39.4 ng/ml (Fig. 4). Culturing on collagen type I and fibronectin also increased the production of GRO‐α and HGF, but not IL‐8, significantly by PDMCs. Thus, culturing on laminin‐coated plates enhanced the paracrine functions of PDMCs the most.

### Laminin enhances the anti‐apoptotic effects of PDMC‐CM on cardiomyocytes

**Figure 5:**
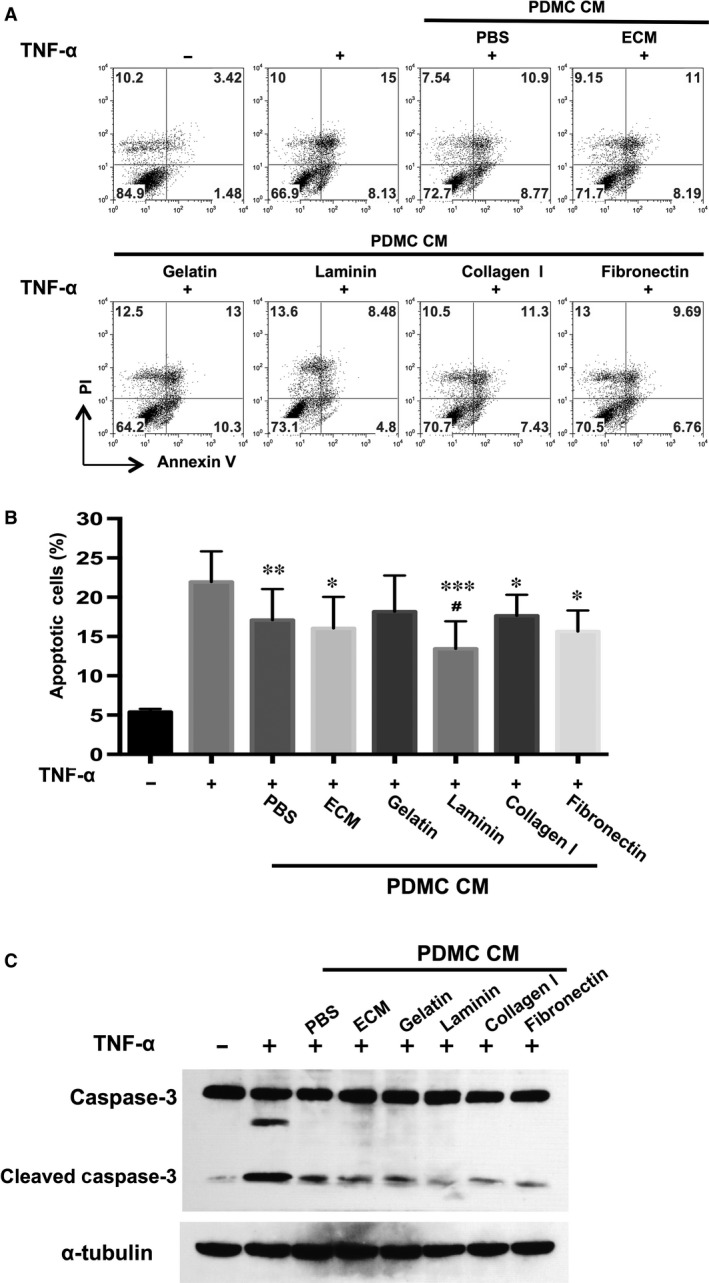
CM from laminin‐cultured PDMCs possesses an enhanced capacity to suppress mCardio apoptosis. (**A**) mCardios were treated with treated with TNF‐α alone or mixed with CM from PDMCs cultured on uncoated plates (PDMC‐PBS‐CM), plates coated with various proteins as indicated including laminin (PDMC‐lam‐CM) or mouse cardiomyocyte CM (mCardio‐CM). After 48 hrs, cardiomyocytes were stained for annexin V and propidium iodine (PI) and analysed by flow cytometry. Representative graphs and (**B**) quantitative results are shown. (**C**) Activated/cleaved caspase‐3 in TNF‐α‐treated mCardios after the addition of PDMC‐CM from uncoated plates (PBS) or plates coated with various proteins as indicated was assayed by Western blotting. Data are shown as the mean ± S.E.M. of three independent experiments. *P < 0.05; **P < 0.01; and ***P < 0.005 compared to TNF‐α only; #*P* < 0.05 compared to PDMC‐PBS‐CM.

To verify whether the laminin‐enhanced paracrine functions of PDMCs were functionally relevant, we collected the CM from PDMCs cultured as usual (uncoated; PDMC‐CM) or cultured on various ECM protein‐coated plates and treated TNF‐α‐stimulated cardiomyocytes with the CM. We found that CM collected from PDMCs cultured on laminin‐coated plates (PDMC‐lam‐CM) was more effective at reducing TNF‐α‐induced apoptosis in cardiomyocytes than CM from PDMCs cultured on plates coated with the other ECM proteins (Fig. 5A and B). Moreover, PDMC‐lam‐CM also inhibited the formation of the activated form of caspase‐3 (Fig. 5C). These data indicated that introducing laminin into the *in vitro* culture environment modulates the paracrine functions of PDMCs in a manner that is functionally relevant.

### PDMC secretion of GRO‐α, HGF and IL‐8 but not IL‐6 can be modulated by laminin through αvβ3 integrin

**Figure 6:**
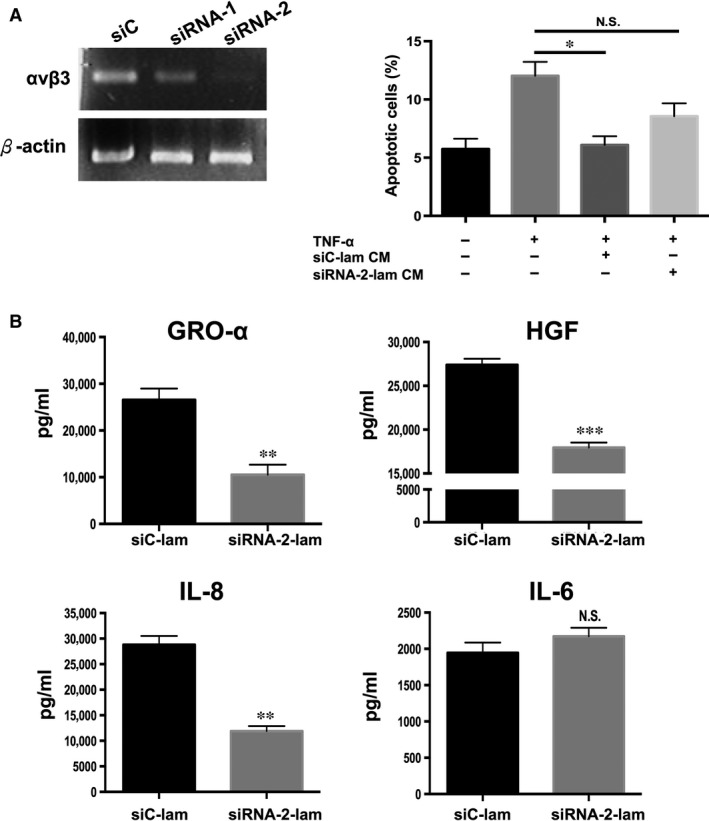
Laminin enhances PDMC paracrine functions through αvβ3 integrin/CD61. (**A**) Gene expression analysis by RT‐PCR following knockdown in PDMCs of αvβ3 integrin expression by small interfering RNA (siRNA) specific for the gene (siRNA‐1 and siRNA‐2) and si‐non‐target (control). Subsequent experiments were performed with siRNA‐2. (**B**) ELISA of GRO‐α, IL‐8 and HGF secretion by PDMCs cultured in laminin‐coated plates after knockdown of αvβ3 integrin expression. (**C**) mCardios treated with TNF‐α alone or mixed with CM from PDMCs after knockdown of αvβ3 integrin expression and cultured on laminin‐coated plates (siRNA‐2‐lam‐CM). After 48 hrs, cardiomyocytes were stained for annexin V and PI and analysed by flow cytometry. Quantitative results are shown, and the data are presented as the mean ± S.E.M. of three independent experiments. *P < 0.05; **P < 0.01; and ***P < 0.005 compared to siC‐lam; N.S., not significant.

To investigate whether laminin can modulate PDMC paracrine functions through αvβ3 integrin/CD61, an integrin highly expressed on MSCs and known to have high affinity for laminin, we used siRNA to reduce αvβ3 integrin expression in PDMCs (Fig. 6A) and then assayed for various secreted factors. When the expression of αvβ3 integrin was silenced, it significantly reduced the protein secretion of GRO‐α from 26.5 to 10.5 ng/ml, HGF from 27.4 to 17.9 ng/ml and IL‐8 from 28.8 to 11.9 ng/ml even after culturing PDMCs on laminin‐coated plates, but IL‐6 levels were relatively unchanged (Fig. 6B). Moreover, CM collected from PDMCs cultured on laminin with knockdown of αvβ3 integrin (siRNA‐2‐lam‐CM) was not able to rescue cardiomyocytes from TNF‐α‐induced apoptosis, in contrast with CM collected from PDMCs with non‐target (negative control) gene knockdown (siC‐lam‐CM; Fig. 6C).

### Laminin promotes PDMC secretion of multiple factors through JNK, for GRO‐α and IL‐8 secretion, and PI3K/AKT, for HGF secretion

**Figure 7:**
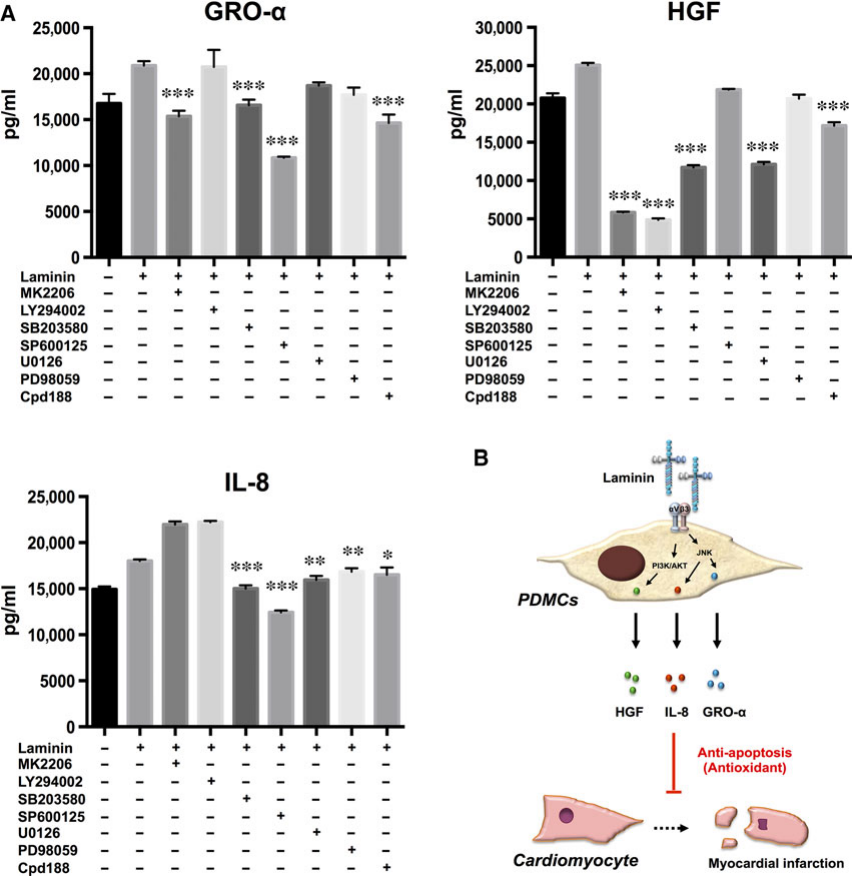
Laminin promotes the secretion of paracrine factors from PDMCs with specific involvement of the signalling pathways of p38, for GRO‐α and IL‐8 secretion, and PI3K/AKT, for HGF secretion (**A**) Secretion of GRO‐α, HGF and IL‐8 by PDMCs was detected by ELISA after treatment with inhibitors of AKT (MK2206, 10 μM), PI3K (LY294002, 10 μM), p38 (SB203580, 20 μM), JNK (SP600125, 20 μM), MEK1/2 (U0126, 20 μM), MEK‐1 (PD98059, 20 μM) and STAT3 (Cpd188, 5 μM) for 48 hrs. All results are shown as the mean ± S.E.M. of three independent experiments. *P < 0.05; **P < 0.01; and ***P < 0.005 compared to laminin only. (**B**) Schematic diagram of the suppression of cardiomyocyte apoptosis and ROS production along with the up‐regulation of antioxidant enzymes by PDMCs through secretion of GRO‐α, HGF and IL‐8, which can be enhanced through the laminin/αvβ3 integrin axis.

Our finding of a single integrin being involved in the up‐regulation of the secretion of multiple distinct paracrine factors was unexpected. To elucidate how this is mediated, we determined the signalling pathways that are involved in this process. We therefore treated laminin‐cultured PDMCs with inhibitors of major signalling pathways (Fig. 7A) and found that JNK inhibition (with SP600125) led to the most significant decreases in GRO‐α and IL‐8 secretion, from 20.9 to 10.8 ng/ml and 18.0 to 12.4 ng/ml, respectively. Inhibition of the PI3K/AKT pathways (with MK2206 and LY294002, respectively), the MAP kinase pathways of MEK1/2 or MEK‐1 (with U0126 and PD98059, respectively), or STAT3 (with Cpd188) had little or no effect. On the other hand, PDMC secretion of HGF was strongly affected by inhibition of either the PI3K (with MK2206) or AKT pathway (with LY294002), from 25.0 to 5.8 and 4.9 ng/ml, respectively, but was minimally affected by inhibition of other pathways. Collectively, these results demonstrated that laminin promoted the PDMC secretion of multiple factors mainly through JNK, for GRO‐α and IL‐8 secretion, and PI3K/AKT, for HGF secretion. Thus, our findings demonstrate that the laminin/αvβ3 integrin axis promotes the PDMC secretion of GRO‐α, IL‐8 and HGF through multiple but specific signalling pathways (Fig. 7B).

## Discussion

Our findings demonstrate that factors secreted by PDMCs suppress apoptosis and reduce ROS production in TNF‐α‐induced cardiomyocyte death. Moreover, these beneficial paracrine effects can be augmented in an *in vitro* setting by culturing PDMCs on laminin, which engages αvβ3 integrin expressed on these foetal‐stage MSCs. Coupled with our recent results on the angiogenic and possible transdifferentiation of PDMCs in murine and porcine models of acute MI 9, our current data indicate that these foetal‐stage MSCs may be good candidates for the treatment of IHD.

In this study, we found that PDMCs secrete high levels of GRO‐α, HGF and IL‐8, which have roles in reducing apoptosis and ROS production; the levels of these paracrine factors can be further up‐regulated through modulation of the laminin/αvβ3 integrin axis. GRO‐α and IL‐8 are members of the CXC chemokine family, and they share a common receptor: CXCR2 22. Our data show that IL‐8 is more potent at suppressing apoptosis than GRO‐α; this may be because IL‐8 is a ligand for two receptors, CXCR1 and CXCR2, whereas GRO‐α is recognized only by CXCR2. Both CXCR1 and CXCR2 have been reported to regulate cell death by reducing apoptosis and necrosis 23. CXCR2 is widely expressed on normal and tumour cells, and although we did not investigate the involved downstream signalling pathways, there are a number of reports in the literature demonstrating that the engagement of this receptor promotes cell survival and regulates anti‐apoptotic genes 22, 24, including suppression of TNF‐α‐induced apoptosis and ROS production 25, 26, 27. Interestingly, our data also demonstrate that the laminin/αvβ3 integrin axis‐mediated up‐regulation of these two chemokines is mediated mainly through JNK, whereas the up‐regulation of HGF is mainly mediated through PI3K/AKT (Fig. 7A). HGF, which is highly secreted by many types of stromal cells including MSCs, is known to be involved in multiple cellular processes, including proliferation and anti‐inflammation 28, 29, 30. Engagement of its only receptor, c‐MET, leads to the activation of four major signalling pathways, and the PI3K/AKT pathway is commonly involved in the ligand's proliferative and anti‐apoptotic functions in both normal and neoplastic cells 31. In contrast, IL‐6 – a cytokine also highly secreted by PDMCs and MSCs 32, 33, 34 – did not reduce TNF‐α‐induced apoptosis and was not further up‐regulated through the laminin/αvβ3 integrin axis. These results are in line with previous findings that the IL‐6 inhibition of caspase‐3 is mediated through TGF‐β but not TNF‐α 35. IL‐6, a pleomorphic cytokine, is highly linked with inflammation and disease, especially in the cardiac system, and involves ERK signalling 36, 37. The lack of an IL‐6 response in PDMC‐mediated cell therapy clearly can be beneficial in the setting of IHD and supports the specificity of laminin/αvβ3 integrin modulation and lack of ERK activation of PDMC paracrine functions. However, such speculation would require further research for substantiation.

In the endogenous environment of the stem cell niche, a number of ECM proteins have been found to regulate crucial stem cell functions, including proliferation, migration and lineage commitment 13, 15. One of the most active areas of research in stem cell therapy is how to mimic the *in vivo* niche for more efficient induction of lineage commitment *ex vivo* for the ultimate goal of improving therapeutic efficacy. Our data demonstrate that through the *in vitro* use of laminin, the paracrine effects of PDMCs can be enhanced with functional relevance. The use of ECM gel, which is more complex – containing laminin, collagens, heparan sulphate proteoglycan and other minor components – and presumed to be more reflective of *in vivo* environments, surprisingly was not as effective as using laminin only, a single type of ECM protein. It may be that in order to enhance the secretion of GRO‐α, HGF and IL‐8 by PDMCs over constitutive levels, a very potent agent is needed; thus, purified, single‐agent proteins rather than a mixed compound – in which one single component would be at a lower concentration – may be more effective. Our results showing that knockdown of a specific integrin can reverse the effects of laminin may offer some indirect support. Moreover, these results also demonstrate that in MSCs/PDMCs, laminin, which binds to multiple integrin receptors 38, 39, does bind with high affinity to the αvβ3 integrin 16, 18. Our findings on the multiple signalling pathways involved downstream in the laminin induction of PDMC paracrine function are in line with emerging reports on ECM/integrin interactions in cell ‘sensing’ of the microenvironment to modulate intracellular signalling pathways and coordinate cellular responses 40. Overall, our findings on the potent and specific effects of the laminin/αvβ3 integrin axis further support the importance of the microenvironment in modulating stem cell functions and highlight the capacity for the manipulation of *in vitro* culture conditions to affect stem cell function.

## Conflict of interests

The authors declare no conflict of interests.
